# PSMA PET/CT Incidental Detection of Tumor Thrombus From Unsuspected Renal Cell Carcinoma and Comparison with FDG PET/CT

**DOI:** 10.1155/crra/8358399

**Published:** 2025-10-29

**Authors:** William Y. Raynor, Stephen J. Sozio, Anthony Yudd, Tina Mayer, Jeffrey S. Kempf

**Affiliations:** ^1^Department of Radiology, Rutgers Robert Wood Johnson Medical School, New Brunswick, New Jersey, USA; ^2^Division of Medical Oncology, Department of Medicine, Rutgers Cancer Institute, Rutgers Robert Wood Johnson Medical School, New Brunswick, New Jersey, USA

**Keywords:** ^18^F-DCFPyL, ^18^F-fluorodeoxyglucose, positron emission tomography, PSA, radioligand therapy, RCC

## Abstract

Clear cell renal cell carcinoma (ccRCC) is typically prostate-specific membrane antigen (PSMA)-avid, likely related to PSMA expression in the tumor neovasculature, suggesting a potential role for evaluation by PSMA PET/CT. We describe a 77-year-old patient with prostate cancer who was incidentally found to have ccRCC on imaging with PSMA PET/CT, with subsequent staging by FDG PET/CT. He was diagnosed with prostate cancer 17 years prior and treated with radical prostatectomy and radiation therapy within a year of diagnosis. Biochemical recurrence with PSA of 1.0 ng/mL prompted imaging with PSMA PET/CT, which showed an unexpected finding of abnormal uptake within the right renal vein and inferior vena cava (IVC), suggesting possible tumor thrombus (SUVmax 8.8), with mild uptake corresponding to a suspected right renal mass. In addition, there was a PSMA-avid right paratracheal nodal metastasis measuring 3.3 cm (SUVmax 8.4). Subsequent FDG PET/CT again showed the renal mass, tumor thrombus (SUVmax 3.6), and only low-level uptake in the right paratracheal mass (SUVmax 2.3). Right nephrectomy was performed, confirming the presence of ccRCC in the right kidney with tumor thrombus extending to the right renal vein and IVC. FDG PET/CT restaging showed no recurrence in the nephrectomy bed and a stable FDG-avid right paratracheal mass. After stereotactic body radiation therapy (SBRT) directed to the right paratracheal mass, follow-up PSMA PET/CT showed decreased uptake (SUVmax 4.8), suggesting its usefulness for detecting and monitoring ccRCC.

## 1. Introduction

Radiotracers used in prostate-specific membrane antigen (PSMA) positron emission tomography/computed tomography (PET/CT) include ^68^Ga-PMSA-11, ^18^F-DCFPyL, and ^18^F-rhPSMA-7.3, which bind to the transmembrane protein PSMA. PSMA PET/CT is now in routine clinical use for the detection of prostate cancer, specifically for staging, assessment of biochemical recurrence, and planning radionuclide therapy [1]. However, several nonprostatic solid tumors such as clear cell renal cell carcinoma (ccRCC) also demonstrate high levels of PSMA associated with the tumor neovasculature, resulting in high PSMA uptake and potentially indicating a role for PSMA PET/CT in the evaluation of additional tumor types [2–5]. We present a case of metastatic ccRCC with associated tumor thrombus which was incidentally detected on ^18^F-DCFPyL PET/CT and later imaged by ^18^F-fluorodeoxyglucose (FDG) PET/CT, helping to direct the course of treatment.

## 2. Case Presentation

**Figure 1:**
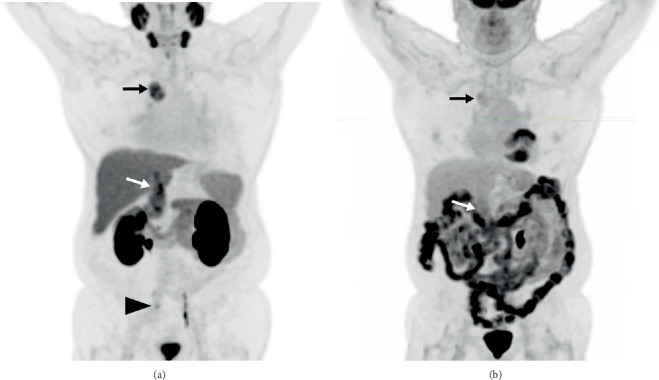
Maximum intensity projections (MIPs) of the baseline ^18^F-DCFPyL PET/CT (a) and FDG PET/CT (b) studies. In addition to the pelvic nodal metastasis from prostate cancer noted on PSMA PET (a, arrowhead), additional sites of abnormal uptake ultimately led to a new diagnosis of ccRCC. PSMA PET/CT demonstrated greater radiotracer uptake than FDG PET/CT at both the right paratracheal metastasis (a and b, *black arrows*) and tumor thrombus (a and b, *white arrows*).

**Figure 2:**
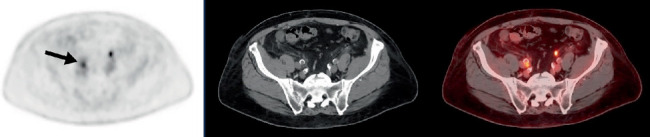
A single suspected nodal metastasis from prostate cancer was present in the pelvis, with abnormal PSMA uptake (*black arrow*; SUVmax 4.0) localizing to a 4-mm lymph node posterior to the right external iliac artery.

**Figure 3:**
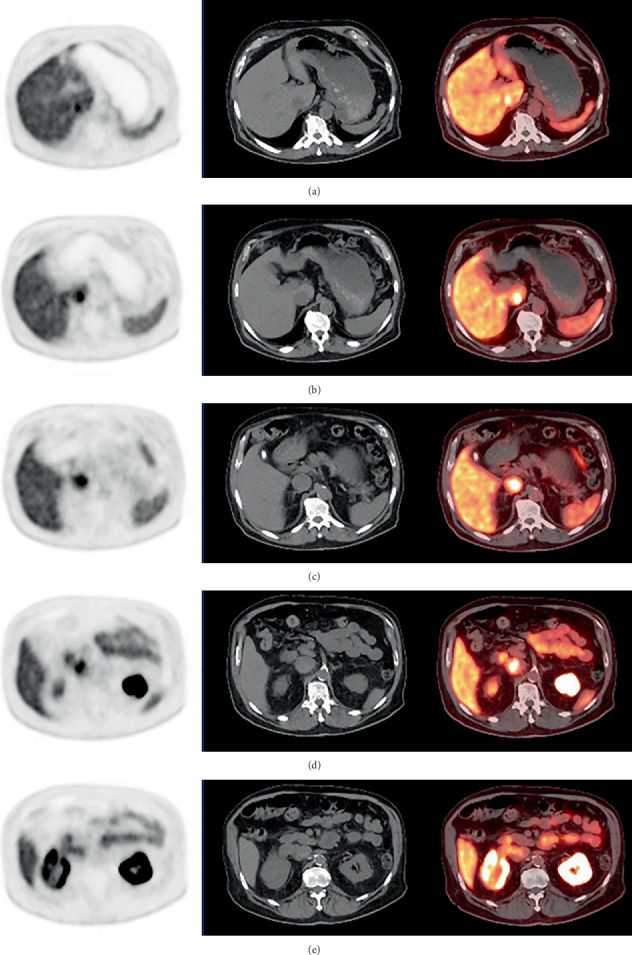
Axial PET, CT, and fused PET/CT images from the same baseline PSMA PET/CT (a–e) shown in Figure 1a with PSMA-avid tumor thrombus extending superiorly to the intrahepatic IVC (a) and inferiorly to the right renal vein (e). The suspected primary right renal mass appears relatively photopenic compared to the remainder of the kidney (e).

**Figure 4:**
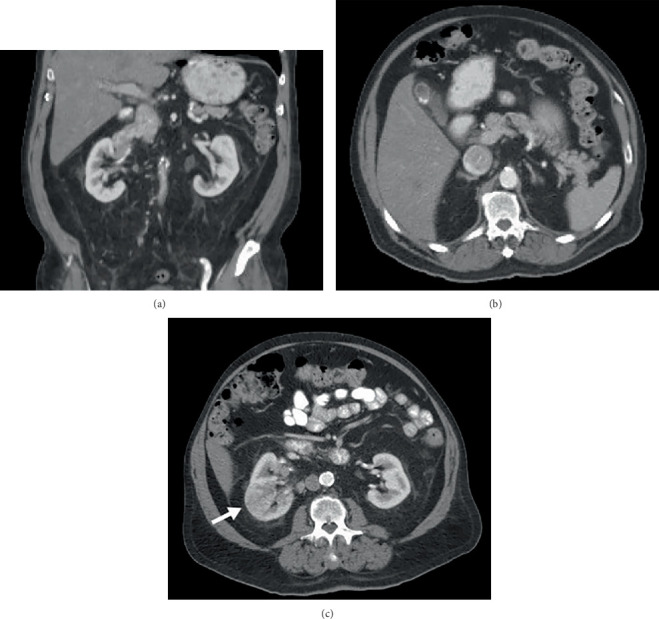
Coronal contrast-enhanced CT (CECT), performed before PSMA PET/CT, in which a right renal mass appears to extend to the right renal vein and IVC (a). The filling defect within the suprarenal IVC (b) and the right renal mass (c, *white arrow*) are also appreciated on axial images.

**Figure 5:**
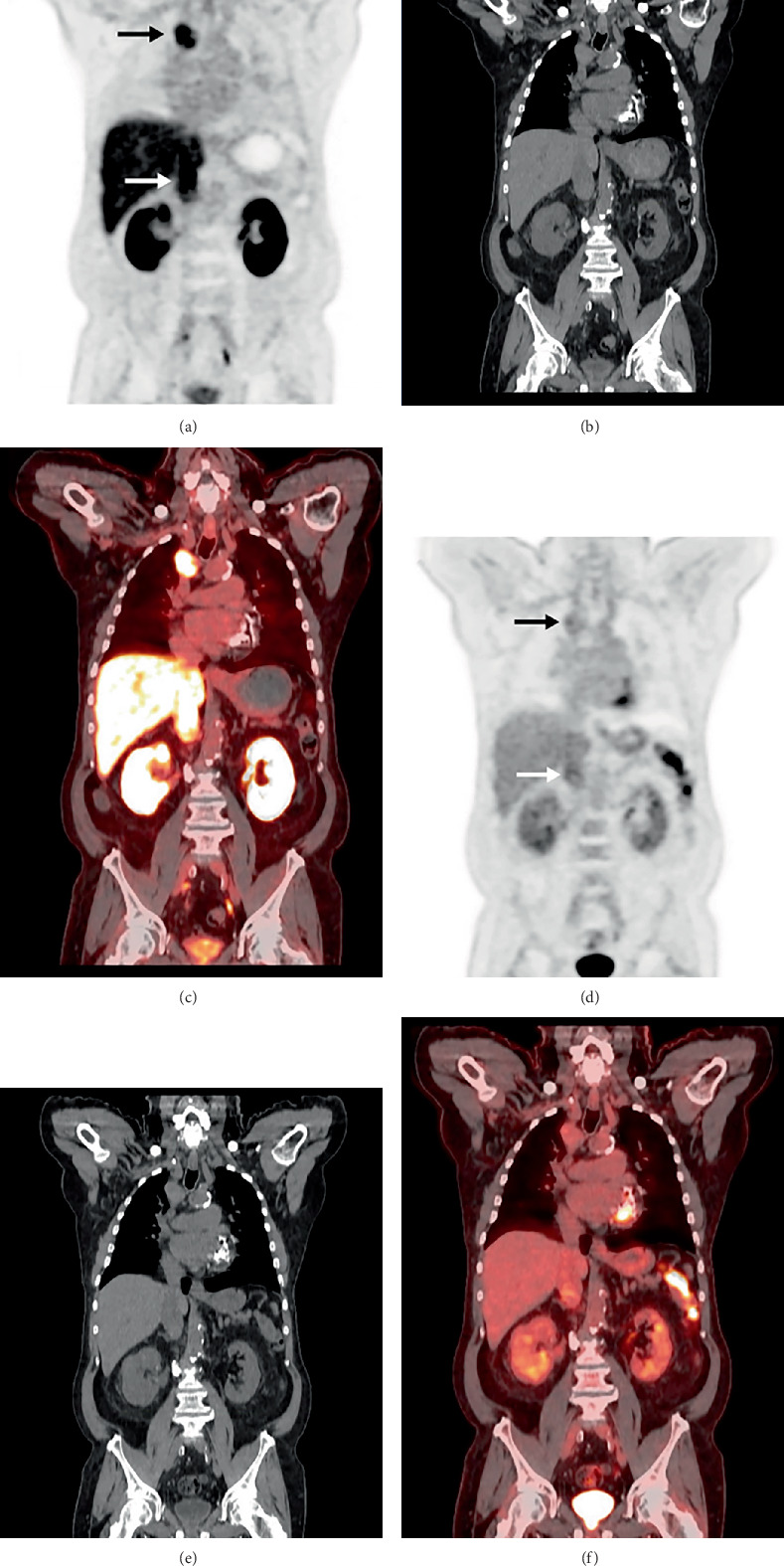
Coronal PET, CT, and fused PET/CT images from the same baseline ^18^F-DCFPyL PET/CT (a–c) and FDG PET/CT (d–f) studies shown in Figure 1. The right paratracheal metastasis demonstrated an SUVmax of 8.4 on ^18^F-DCFPyL PET/CT (a, *black arrow*), compared to an SUVmax of 2.3 on FDG PET/CT (d, *black arrow*). Similarly, the tumor thrombus in the IVC had higher ^18^F-DCFPyL uptake (SUVmax 8.8; a, *white arrow*) compared to that of FDG (SUVmax 3.6; d, *white arrow*).

^18^F-DCFPyL PET/CT was performed for assessment of biochemical recurrence of prostate cancer in a 77-year-old patient. At the time of initial prostate cancer diagnosis, his PSA was 6.2 ng/mL. He subsequently underwent radical prostatectomy, which revealed adenocarcinoma with Gleason score 7 (3 + 4) and periprostatic extension. Radiation therapy was performed to the prostate bed, after which PSA levels were undetectable (< 0.01 ng/mL). Approximately two decades later, he developed biochemical recurrence and underwent subsequent PSMA PET/CT with PSA of 1.0 ng/mL at the time of imaging (Figure 1). Abnormal PSMA uptake was present in the right iliac region, suspected to represent metastatic prostate cancer (Figure 2). There was additional PSMA localization to the region of the right renal vein and inferior vena cava (IVC), compatible with suspected tumor thrombus (SUVmax 8.8) (Figure 3). There was a suspected right renal mass with only mild PSMA uptake, not detected on a recent outside contrast-enhanced CT of the abdomen and pelvis, where the findings of a renal neoplasm extending to the renal vein and IVC were only appreciated in retrospect (Figure 4). An additional PSMA-avid right paratracheal nodal metastasis measuring 2.7 × 3.3 cm was also present (SUVmax 8.4), which was of indeterminate etiology (Figure 5). The findings involving the right kidney and mediastinum were purely incidental as the patient did not present with any related symptoms. FDG PET/CT was then performed for staging of the renal mass, which showed the right paratracheal nodal metastasis with only low-level uptake (SUVmax 2.3), lesion in the upper pole of the right kidney (SUVmax 2.5), and tumor thrombus extending to the IVC (SUVmax 3.6). Therefore, this was considered Stage IV (T3cN0M1) by the latest American Joint Committee on Cancer (AJCC) staging criteria [6].

**Figure 6:**
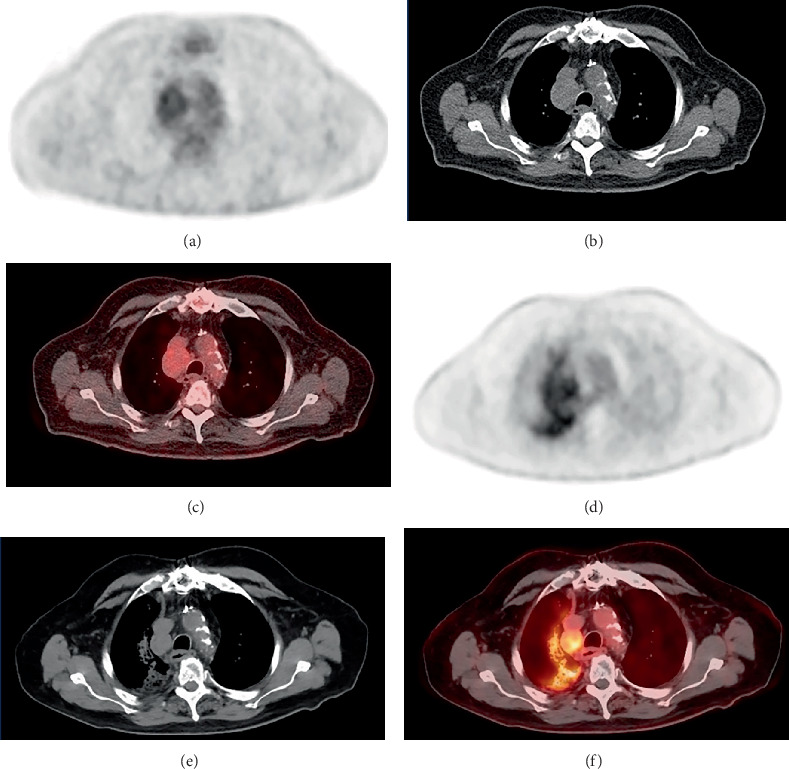
Restaging after nephrectomy showed stable FDG uptake by the mediastinal metastasis as demonstrated on axial PET/CT images (a–c). The patient underwent further treatment with SBRT. Follow-up PSMA PET/CT (d–f) was obtained to restage prostate cancer approximately 9 months after the initial study which showed the pelvic prostate cancer metastasis and incidentally identified the renal mass. The decrease in size from 3.5 to 2.7 cm as well as the decrease in PSMA uptake (SUVmax 4.8, previously 8.4) associated with the right paratracheal ccRCC metastasis suggested a partial response to therapy, although the precise implications of changes in PSMA uptake warrant further investigation.

Right nephrectomy revealed ccRCC in the right kidney with tumor thrombus extending to the right renal vein and IVC, with fine needle aspiration of the right paratracheal mass confirming the presence of a ccRCC metastasis. Repeat FDG PET/CT after the nephrectomy showed no evidence of local recurrence in the surgical bed as well as no significant change in the FDG-avid paratracheal metastasis. Several months later, after the administration of stereotactic body radiation therapy (SBRT) to the right paratracheal mass (40 Gy in five fractions), follow-up PSMA PET/CT was performed, which showed decreased uptake by the paratracheal ccRCC metastasis after therapy (Figure 6). Based on these findings, continued imaging surveillance was planned with systemic therapy for ccRCC to be considered only in the event of imaging evidence of disease progression. Surveillance was also planned for the management of his prostate cancer given prolonged time to recurrence, slow PSA rise, and subcentimeter pelvic lymph node disease. Several years since the biochemical recurrence and without treatment directed for prostate cancer, his PSA has nonetheless been fluctuating between 1.4 and 2.1 ng/mL, most recently measured at 1.7 ng/mL.

## 3. Discussion

Renal cell carcinoma (RCC) represents approximately 3% of cancer in adults, most often being diagnosed incidentally on imaging [7, 8]. RCC has three main histopathologic subtypes: clear cell carcinoma (ccRCC) (70%–80%), papillary RCC (10%–15%), and chromophobe RCC (5%) [7, 9]. A significant proportion of patients are found to have metastatic disease at the time of diagnosis, with an additional subset of patients developing metastases after curative surgery [10]. Therefore, the challenge of managing RCC necessitates advanced diagnostic tools for precise staging and treatment planning.

Although FDG PET/CT is widely used in other malignancies, it has limited value in RCC due to renal excretion of FDG and variable GLUT-1 expression in RCC cells [10]. Therefore, there is growing interest in identifying new PET tracers for imaging of RCC. For example, the multicenter ZIRCON trial recently announced promising results by demonstrating high sensitivity and specificity of ^89^Zr-girentuximab for detection of ccRCC [11]. Meanwhile, PSMA PET/CT has emerged as another potential molecular imaging technique for the evaluation of RCC [12]. Al-Ibraheem et al. have noted the utility of PET/CT imaging with both FDG and PSMA to detect synchronous malignancies [13]. As a tumor marker, PSMA is found in endothelial cells of the tumor neovasculature in ccRCC, which tends to highly express vascular endothelial growth factor (VEGF) [14, 15]. PSMA PET/CT effectively detects intrarenal and metastatic ccRCC lesions, surpassing conventional imaging for metastases, although CT remains superior for small pulmonary metastases [16–18]. Among 37 RCC patients assessed in a 2024 study by Aggarwal et al., ^68^Ga-PSMA-11 PET/CT was superior to CT in detecting ccRCC tumor thrombi and bone marrow metastases [19]. A systematic review by Jóźwik-Plebanek et al. concluded that renal versus hepatic excretion of PSMA PET tracers influences their diagnostic sensitivity, with radiotracers undergoing hepatic excretion showing slightly better sensitivity for intrarenal RCC lesions [10].

The use of PSMA PET/CT for detecting metastatic ccRCC has shown promise across several previously published studies. Initially reported in 2014 by Demirci et al., the authors presented a case in which PSMA PET/CT revealed more metastatic sites in a patient with ccRCC compared to FDG PET/CT, demonstrating higher sensitivity [20]. Rowe et al. further supported this conclusion with a case series, showing that PSMA PET/CT detected more metastases than conventional imaging with improved sensitivity for small lesion detection [21]. Since then, multiple prospective and retrospective studies by other research groups also showed high accuracy of PSMA PET/CT for detecting ccRCC metastases while noting decreased tumor-to-background uptake of primary RCC lesions due to high physiologic renal activity [16, 17, 22–24]. Furthermore, the prospect of treating metastatic ccRCC with ^177^Lu-labeled PSMA ligands as part of a theranostic approach is under active investigation [2]. Preliminary data regarding the use of radioligand therapy using PSMA for treatment of ccRCC show early potential, although rapid radiotracer washout from ccRCC lesions appears to limit therapeutic efficacy [25]. Continued investigation to refine diagnostic and therapeutic applications of PSMA PET/CT for RCC is necessary to determine the optimal use of this new approach.

In our case, while both the ccRCC primary tumor and mediastinal metastasis demonstrated PSMA and FDG uptake, PSMA uptake was significantly higher at both sites, concordant with previous studies which noted higher SUVs in RCC using PSMA tracers compared to FDG [26]. In addition, follow-up PSMA PET/CT suggested partial response of the paratracheal mass to radiotherapy, although future studies are needed to determine the correlation between uptake and treatment response. These observations further support the utility of PSMA PET/CT in identifying and monitoring ccRCC, suggesting evaluation of ccRCC as a possible future clinical indication for PSMA PET/CT.

## 4. Conclusion

PSMA PET/CT detects ccRCC with high sensitivity, often surpassing that of CT and MRI, as illustrated by the case presented here. Diagnostic information from PSMA PET/CT can play an important role in directing the course of therapy; however, early efforts to use PSMA-based radioligand therapy in ccRCC have been limited by rapid tracer washout from the sites of disease. Further research is warranted to validate ccRCC staging and monitoring with PSMA PET/CT and to determine the future role, if any, radionuclide therapy with PSMA may play in ccRCC treatment.

## Data Availability

Data sharing not applicable to this article as no datasets were generated or analyzed during the current study.
